# Linking the Composition of Bacterial and Archaeal Communities to Characteristics of Soil and Flora Composition in the Atlantic Rainforest

**DOI:** 10.1371/journal.pone.0146566

**Published:** 2016-01-11

**Authors:** Julia Elidia Lima-Perim, Emiliana Manesco Romagnoli, Francisco Dini-Andreote, Ademir Durrer, Armando Cavalcante Franco Dias, Fernando Dini Andreote

**Affiliations:** 1 Department of Soil Science, ESALQ/USP, University of São Paulo, Piracicaba, Brazil; 2 Microbial Ecology Group, Genomic Research in Ecology and Evolution in Nature (GREEN), Groningen Institute for Evolutionary Life Sciences (GELIFES), University of Groningen, Groningen, The Netherlands; Medical University Graz, AUSTRIA

## Abstract

The description of microbiomes as intrinsic fractions of any given ecosystem is an important issue, for instance, by linking their compositions and functions with other biotic and abiotic components of natural systems and hosts. Here we describe the archaeal and bacterial communities from soils of the Atlantic Rainforest in Brazil. Based on the comparison of three areas located along an altitudinal gradient—namely, Santa Virginia, Picinguaba and Restinga—we detected the most abundant groups of Bacteria (*Acidobacteria* and *Proteobacteria*) and Archaea (*Thaumarchaeota*, *Crenarchaeota* and *Euryarchaeota*). The particular composition of such communities in each of these areas was first evidenced by PCR-DGGE patterns [determined for Bacteria, Archaea and ammonia-oxidizing organisms—ammonia-oxidizing archaea (AOA) and bacteria (AOB)]. Moreover, sequence-based analysis provided a better resolution of communities, which indicated distinct frequencies of archaeal phyla and bacterial OTUs across areas. We found, as indicated by the Mantel test and multivariate analyses, a potential effect of the flora composition that outpaces the effect of soil characteristics (either physical and chemical) influencing the assembly of these microbial communities in soils. Our results indicate a collective role of the ecosystem underlying observed differences in microbial communities in these soils. Particularly, we posit that rainforest preservation also needs to take into account the maintenance of the soil biodiversity, as this is prompted to influence major processes that affect ecosystem functioning.

## Introduction

The Brazilian Atlantic Rainforest is a well-known reservoir for biodiversity, hosting approximately 2% of the total species worldwide [1,2]. This biome is characterized as a mosaic of ecosystems, with distinct compositions of fauna and flora, driven by the variability of soils, topography and climate conditions along a broad area of occurrence in Brazil [3,4]. The maintenance of such biome is largely based on the high efficiency of nutritional supply that takes place at the base of the food web, i.e. in metabolic processes exerted to a large extent by the dwelling soil microbial communities [5–7].

High levels of alpha- and beta-diversities have been reported in microbial community assessments in rainforest studies [4,8–9]. These communities are known to be highly responsive to land-use changes, as, for instance, by the conversion of natural forest areas into agricultural fields [3,9,10]. Moreover, microbial communities differ along natural gradients that take place within the forest mosaic (e.g. altitudinal gradients [11,12]), diverging in composition according to local fauna and flora compositions [13,14], and even along gradients of natural forestry recovery [15]. These variations are commonly correlated with changes in soil pH [8,16,17]. In the Atlantic Rainforest, a detailed view of the influence of soil edaphic properties on the composition of microbial communities has been provided [4], highlighting for pH, organic matter (OM), phosphorus (P) and cation (Ca and Mg) concentrations as the main soil properties influencing the levels of microbial community composition and diversity in these soils.

In a broader view, the variations in soil microbial communities are thought to occur within a normal operating range [18]. However, specific fractions of the soil microbiome are more responsive to environmental conditions than others, as for instance, those observed for ammonia-oxidizing archaea (AOA) and ammonia-oxidizing bacteria (AOB) [19,20]. Notably, these functional communities have been broadly studied in different soil systems in order to identify critical environmental parameters affecting their composition and function in natural environments [21,22], and even in the face of anthropogenic impacts [23–25].

In this study, we focused on providing the assessment of the composition, abundance and characteristics of two distinct microbial domains (i.e. Bacteria and Archaea) in soils collected along a natural altitudinal gradient located in the Brazilian Atlantic Rainforest. We expected that the distinct comparison of three micro-climatic areas would allow us to disentangle the relationships among soil characteristics, flora composition and microbial communities. To achieve that, total bacterial and archaeal communities were profiled, and the specific communities of AOA and AOB, known to be sensitive to environmental shifts, were targeted in these soils.

## Material and Methods

### Ethical Statement

The authorization for sampling the studied areas is regulated by the Brazilian Ministry of Environment. It issued permission to these sites to the group leader and to the University of Sao Paulo (authorization number 49476–1). We also confirm that this field study did not involve endangered or protected species.

### Sampling sites, physical and chemical properties of soils

Soil samples were collected in February 2011, along an altitudinal gradient in the Atlantic Rainforest located in the North of São Paulo State, Brazil. Soils in this area are mostly classified as Yellow-Red Oxisoils, occurring under a dense ombrophylous forest in a region that does not have dry seasons. At the sampling time, average air temperature was consistent in all threes sites, ranging from 18°C to 23°C. All samples were collected under the native forest largely composed by high plants (30 to 50 m height).

Three distinct areas were selected along a gradient of altitude in the preserved state park Serra do Mar. The first site, Restinga (23°21' S and 44°51' W), was located at sea level (0 ± 5 m); followed by the site Picinguaba (23°36' S and 44°81' W), at an altitude of 50 to 100 m; and the highest site, Santa Virginia (23°17’ S and 45°11’ W), reaching up to 900 to 1,000 m. In each area, four gridded plots were established (10 m x 10 m), approximately 100 m apart from each other. In each plot, litter materials were removed and ten soil cores (5-cm diameter x 10-cm depth) were taken using sterile techniques, to represent one composite sample per plot. The soil layer sampled (top 10 cm) was chosen since it encompasses the large proportion of the soil biomass where most of the chemical transformations take place (the ‘active soil layer’). Each sample was placed in a sterile plastic bag, sealed and transported to the laboratory (<24 h). All samples were sieved through a 4.0 mm sieve and stored at -20°C.

For chemical measurements, soil samples were air-dried and sieved through a 100 mesh for determination of organic matter (OM), nitrate (N-NO_3_^-^), ammonium (N-NH_4_^+^), phosphorous (P), potassium (K), calcium (Ca), magnesium (Mg), aluminum (Al), boron (B), copper (Cu), iron (Fe), manganese (Mn), zinc (Zn) and pH (Table 1). Physical (sand:silt:clay % content) (Table 1) and chemical analyses were carried out in collaboration with the Laboratory of Soil Analysis at “Luiz de Queiroz” College of Agriculture (Department of Soil Sciences, ESALQ/USP, Piracicaba, Brazil) according to the method described by [26]. Ammonium and nitrate concentrations were determined using extraction from 10 g of soil in 50 mL of 2 M KCl, according to the method described by [27].

**Table 1:** Soil characteristics at the three sampling sites of the Atlantic Rainforest. Values are mean ± SE; *n* = 4.

Soil characteristic	Santa Virginia	Picinguaba	Restinga
pH (in CaCl_2_)	3.6±0.1	3.6±0.1	3.7±0.1
Organic Matter (g.dm^-3^)	70.3±8.0	63.7±3.9	62.2±4.0
Sand:silt:clay	55.9:12.4:31.7	56.9:11.3:31.8	87.7: 4.6: 7.7
Total P (mg.dm^-3^)	15.3±1.2	13.0±2.5	5.3±0.3
Total S (mg.dm^-3^)	9.7±0.9	13.0±0.6	5.3±0.3
K (mmol_c_*.dm^-3^)	2.0±0.3	1.7±0.2	0.8±0.1
Ca(mmol_c_.dm^-3^)	2.3±0.3	2.3±0.3	1.0±0.0
Mg (mmol_c_.dm^-3^)	2.3±0.3	2.0±0.0	1.0±0.0
Al (mmol_c_.dm^-3^)	23.3±0.7	20.4±0.6	16.5±1.6
H+Al**(mmol_c_.dm^-3^)	146.3±12.9	139.3±3.2	104.7±12.4
Nitrate (μg/g dry soil)	116.6±12.3	125.7±16.4	25.4±4.8
Ammonium (μg/g dry soil)	26.3±4.2	53.7±12.6	36.5±9.7
B (mg.dm^-3^)	0.42±0.0	0.41±0.0	0.17±0.0
Cu (mg.dm^-3^)	0.6±0.2	0.4±0.2	0.1±0.0
Fe (mg.dm^-3^)	303.0±79.3	199.3±6.0	731.5±428.1
Mn(mg.dm^-3^)	3.3±0.6	3.9±1.0	0.7±0.2
Zn (mg.dm^-3^)	1.0±0.1	0.9±0.1	0.7±0.1

*mmol_c_—millimoles of soil charges occupied by the element

**acidic potential of the soil

Additional metadata were added to the soil characterization—i.e. the flora composition per sampling site [28]. Information on the abundance of native plants was obtained by descriptive annual reports submitted to the funding agency (FAPESP—São Paulo Research Foundation) that financed the previous survey [28]. For statistical analyses, the matrix was obtained based on the description of the number of plant from each species within 1 ha of each sampled area (S1 Table).

### DNA extraction from soil samples

DNA was extracted from 0.5 g of initial soil material using the MoBio PowerSoil DNA isolation kit (MoBio Laboratories, Carlsbad, CA, USA), following the manufacturer’s instructions. The purity of obtained DNAs was checked on a 1.5% agarose gel run at 90 V for 1 h in 0.5x Tris-acetate-EDTA (TAE) buffer (20 mM Tris, 10 mM acetate, 0.5 mM EDTA, pH 8.0). The gel was stained with ethidium bromide for 20 min (1.2 mg L^-1^ ethidium bromide in 0.5x TAE buffer). DNA concentrations were estimated using NanoDrop (Thermo Scientific, USA).

### Quantitative real-time PCR (qPCR) of targeted communities

The abundances of Bacteria, Archaea, AOA and AOB were quantified by qPCR targeting the 16S rRNA and the ammonia monooxygenase (*amoA*) gene, respectively. For Bacteria, primers P1 and P2 [29] were used, generating fragments of 193 bp. Archaea were quantified using the primers 340F and 1000R [30], generating fragments of 660 bp. For AOA, primers amo23F [31] and CrenamoA616r48x [32] were used, generating fragments of 624 bp [33]. For AOB, we used a combination of primers, as follows: CTO189fA/CTO189fB, CTO189fC, CTO654r [34] and R1 [35], which generate fragments of 465 bp. Detailed information on cycling conditions is provided in S2 Table. All quantifications, including positive and negative control reactions (free of DNA from samples), have been done in parallel to monitor potential contamination(s).

Quantifications were carried out twice for each of the soil replicates on the Rotor Gene 6000 (Corbett Life Science, Australia). The specificity of amplifications were confirmed by melting-curve analyses, and the expected sizes of the amplified fragments were checked on a 1.5% agarose gel stained with ethidium bromide. Standard curves were obtained using serial dilutions (10^7^ to 10^2^ gene copies μL^-1^) of plasmid containing specific cloned fragments (AOA and AOB), or even environmental PCR products (Archaea and Bacteria), in the case when degenerated primers were used. The potential inhibitory effects of co-extracted humid compounds were checked by spiking the samples with a standard concentration of gene copies and amplifying the resulting mixes. Consistency in Ct values, and consequently in quantification values, validated our approach. To test for statistically significant differences between gene abundances we used the Tukey's test at 5% probability, carried out in the Assistat 7.4 software [36].

### Analyses of bacterial and archaeal communities by PCR-DGGE

Total bacterial communities were amplified using the primers U968_┴_CG and R1378, generating fragments of 410 bp [37]. Archaeal communities were amplified as described [38], with primers Arch21F and Arch958R in the first reaction, followed by a nested amplification with primers Arch340F_┴_GC and Arch519R, obtaining fragments of 937 and 179 bp, respectively. As a molecular marker for AOA and AOB communities, we used a region of the respective 16S rRNA gene specific for the groups of *Thaumarchaeota* and *Beta*-*proteobacteria*, respectively. AOA was assessed using primers crenamoA23f and crenamoA616r [31], which generated fragments of 620 bp. AOB were assessed with primers CTO189f and CTO654r (450 bp fragments) [34]. Details on cycling conditions are provided in S2 Table.

DGGE profiles were generated with the Ingeny Phor-U System (Ingeny International, Goes, The Netherlands). The PCR products (~120 ng per lane) were loaded onto 6% (w/v) polyacrylamide gels for Bacteria, AOA and AOB and 8% for Archaea. Denaturing gradients of 45–65, 30–55, 15–55 and 35–65% Bacteria, Archaea, AOA and AOB, respectively (where 100% denaturant corresponded to 7 M urea and 40% (v/v) deionized formamide). Electrophoresis was performed at a constant voltage of 100 V for 16 h at 60°C. The gels were stained for 60 min in 0.5 x TAE buffer with SYBR Gold (final concentration of 0.5 μg l^-1^; Invitrogen, Brazil). Images of the gels were obtained by densitometry using a laser densitometer StormTM 845 (GE Healthcare, Uppsala, Sweden) and normalized in the GelCompar II software (Applied Maths, Sint-Martens Latem, Belgium), using the unweighted-pair group method with arithmetic mean, rolling-disk background subtraction, and no optimization [39,40]. DGGE patterns were converted into presence/absence matrices based on band profiles using the software Diversity Database (BioRad, Hercules, CA, USA). The matrices were subjected to further analyses. Clustering analysis was conducted by non-metric multidimensional scaling (NMDS), carried out in PAST [41], using the similarity of Jaccard. Additionally, the validation of clusters and separations were based on R-values obtained by analysis of similarity (ANOSIM) run in Primer5 [42].

The correlations between DGGE patterns and environmental parameters were determined in a multivariate approach where DGGE-based matrices were tested against soil physical and chemical properties and against local plant community composition per area. The first analysis conducted in this context was the Mantel test [43], to determine the congruence of sample separation based on microbiological (DGGE patterns) and contextual (soil physics, soil chemistry and vegetation) parameters. Mantel test was conducted in PAST [41].

Multivariate analysis was performed in CANOCO (Canoco 4.5, Biometris, Wageningen, Netherlands), according to [44]. The linear distribution of data, determined by detrended correspondence analysis (DCA), indicated that redundancy analysis (RDA) was the most suitable model that applied to our dataset. Along with the RDA, each portion of the microbial community assessed was compared with the chosen environmental parameter, testing the significance of each parameter by the Monte Carlo test, based on 499 random permutations. It determined the significance of variables (*p* value) and also generated values of lambda-1, which shows the percentage of the total variance explained by each environmental factor individually (Table 2).

**Table 2:** Significance index (*p*) and explanatory percentage (λA) of the selected variables on the patterns of microbial communities—here obtained by PCR-DGGE patterns—as determined by redundancy analysis (RDA).

	Bacterial 16S rRNA		Archaeal 16S rRNA		*amo*A			
					AOA		AOB	
	λA	*p* value	λA	*p* value	λA	*p* value	λA	*p* value
pH	-	-	-	-	-	-	-	-
Organic matter	-	-	-	-	-	-	-	-
P	-	-	-	-	-	-	2.1	0.002
S	-	-	-	-	-	-	-	-
K	-	-	-	-	-	-	-	-
Ca	-	-	-	-	-	-	-	-
Mg	1.4	0.004	-	-	-	-	-	-
Al	-	-	-	-	3.2	0.008	-	-
H+Al	-	-	-	-	0.8	0.032	-	-
Nitrate	-	-	-	-			-	-
Ammonia	1.0	0.024	1.5	0.004	2.7	0.002	1.6	0.030
B	1.4	0.004	1.8	0.002	-	-	-	-
Cu	2.3	0.002	-	-	-	-	-	-
Fe	-	-	-	-			-	-
Mn	-	-	-	-	1.0	0.032	-	-
Zn	-	-	-	-	-	-	-	-
Sand	-	-	1.7	0.002	-	-	-	-
Silt	2.2	0.002	-	-	-	-	-	-
Clay	-	-	-	-	2.2	0.042	2.2	0.002

### Multi-tag 454-pyrosequencing of the bacterial and archaeal 16S rRNA gene

After the assessment of differences in community patterns based on PCR-DGGE profiles, two samples (duplicates) per area were selected and submitted to multi-tag 454-pyrosequencing of the bacterial and archaeal 16S rRNA genes. For Bacteria, the forward primer 520F and a mixture of four reverse primers 802R were used, see [45] for details. A fragment size of 282 bp in length was obtained, amplifying the V4 region (base position 563 to 802) of the bacterial 16S rRNA gene. For Archaea, the primer set ArcF and ArcR was used [10]. A fragment size of 179 bp was obtained, amplifying the V3 region (base position 340 to 519) of the archaeal 16S rRNA gene (S2 Table). All used primers contained specific 8-bp barcode sequences for further demultiplexing of data. Amplicons from all samples were pooled in equimolar concentrations and sequenced at the Helixxa facility (Genomics Service Provider, Campinas, SP, Brazil) on a Roche GS-FLX 454 automated pyrosequencer running the Titanium chemistry.

### Sequence data processing

Raw pyrosequencing data were demultiplexed and processed using the Quantitative Insights Into Microbial Ecology (QIIME) toolkit [46]. In brief, 16S rRNA partial sequences were trimmed using the following parameters: quality score >25, sequence length >100 and <400, maximum homopolymer of six, zero maximum ambiguous bases and zero mismatched bases in the primer. The quality reads were then binned into operational taxonomic units (OTUs) at 97% sequence similarity using uclust [47] followed by selection of a representative sequence. Chimeric sequences were identified using ChimeraSlayer [48] and removed. The taxonomy was assigned to each representative sequence based on comparisons with sequences in the RDP [45]. For all OTU-based analyses, the original OTU was rarified to a depth of 3,249 and 723 sequences per sample for Bacteria and Archaea, respectively (the fewest in a single sample, to minimize effects of sampling effort on the analysis). QIIME was also used to generate weighted/unweighted UniFrac distance matrices [49], a Bray-Curtis distance matrix [50], and alpha-diversity metrics (observed OTUs, Chao1 richness estimator, Shannon diversity, phylogenetic diversity and Goods coverage). The relation between bacterial and archaeal beta-diversities across the different areas was determined by Procrustes analysis, carried out in QIIME using either the weighted or unweighted UniFrac distance matrices as input. The significance of Procrustes transformation was determined by comparing the residual sum of squares after matching (M^2^), which measures the remaining “lack of fit” of one configuration to the other [51] based on a distribution of M^2^ values empirically determined from 10,000 permutations.

We used the 16S rRNA gene reference sequence from *Candidatus* Nitrosoarchaeum koreensis, *Candidatus* Nitrosopumilus maritimus and *Candidatus* Nitrososphaera gargensis to filter and select sequences potentially associated with AOA in our datasets (minimum identity 95%). A similar approach was used for AOB, where we select for sequences closely affiliated to the genera *Nitrospira* and *Nitrosococcus*, as references. The resulting sequences were used to reconstruct a phylogenetic tree, comparing sequences obtained in this study with those close matches present in the RDP database. The phylogeny was reconstructed in Mega 5.0 [52], using the kimura-2 parameter and a neighbor-joining clustering method [53]. All sequencing data from this study were deposited in the MG-RAST database [54] (accession numbers 4570169.3, 4570170.3, 4570171.3, 4570172.3, 4570173.3, 4570174.3, 4570175.3, 4570176.3, 4570177.3, 4570178.3, 4570179.3 and 4570180.3).

## Results

### Soil properties in the sampled areas

Soils in all three sampled areas presented low pH (values ranging from 3.6 to 3.7), high contents of organic matter (ranging from 6.2 to 7.1%), and similar chemical characteristics as those typically presented in low-fertile forest soils. Notably, nitrate contents in forest soils from SV and PC were significantly higher (*p* < 0.01) than those of RE, while the ammonium concentrations were not statistically significant across sites (*p* = 0.162). The soil physical characteristics differed across the sampled areas. In brief, RE soils were sandier, while clayey textures were found in soils from SV and PC (for a full description see Table 1).

### Abundance assessments of targeted communities

The applied quantification systems were efficient to properly determine the abundances of the targeted communities. We found amplification efficiencies of 113%, 90%, 102% and 108% for archaeal and bacterial 16S rRNA genes, AOA and AOB [*amo*A] genes, respectively, with regression values (R^2^) of 0.99 for all systems.

**Fig 1:**
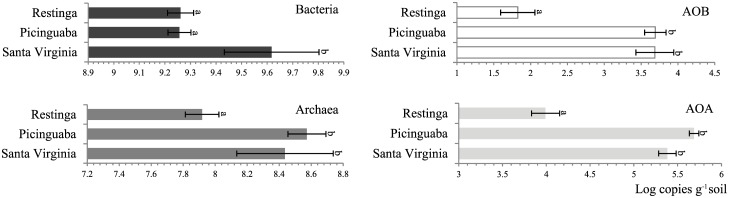
Quantification of targeted fractions—Bacteria, Archaea, AOA and AOB—of the soil microbiome in the three Atlantic Rainforest areas. Bars indicate average values from four replicates, while error bars indicate standard deviations. Bars labeled with the same letter in each graph do not differ statistically according to Tukey’s test (*p* > 0.05).

For the domain Bacteria, soils from SV revealed slightly higher abundances of the 16S rRNA gene than those from RE and PC (*P* < 0.05), with values ranging from 1.84 ×10^9^ to 4.25 ×10^9^ copies of the gene 16S rRNA per gram of soil. For Archaea, lower values were observed, as the abundance of such organisms varied from 1.43×10^8^ to 4.36×10^8^ copies of the 16S rRNA gene per gram of soil, in this case with slightly lower values in soils from RE (*P* < 0.05). The quantification of specific groups involved in ammonia oxidation also revealed differences between areas (*P*<0.05). The abundance of AOA was higher in SV and PC—with values between 2.80×10^4^ and 4.78×10^4^ copies of the archaeal *amoA* gene per gram of soil—than in RE, where the value was 8.03×10^2^ copies per gram of soil (Fig 1). A similar trend was observed for AOB, with higher values for SV and PC, at 5.34×10^4^ and 4.01×10^4^ copies of the specific 16S rRNA gene per gram of soil, respectively, than in RE, at 6.59×10^2^ (Fig 1).

### Fingerprinting the archaeal, bacterial, AOA and AOB communities by PCR-DGGE

**Fig 2:**
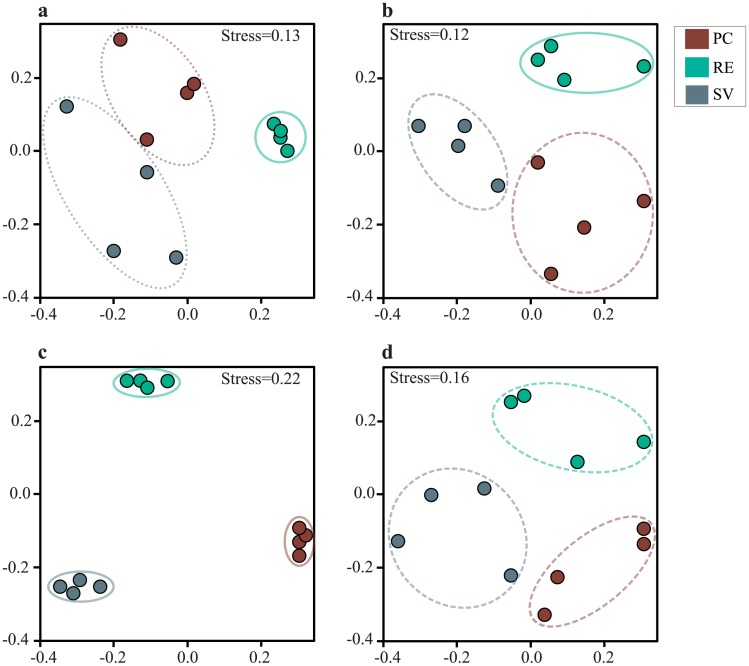
Comparison of community patterns of Archaea (a), Bacteria (b), AOA (c) and AOB (d) along the altitudinal gradient in soils of the Atlantic Rainforest by DGGE—as evidenced by NMDS. Stress values for each community are indicated in the plot. The ANOSIM results (shown in numbers at S3 Table) are indicated by circles, being dotted (R>0.5), dashed (0.5<R<0.75) and full lines (R>0.75).

The groups of Archaea, Bacteria and ammonia oxidizers (AOA/AOB) were evaluated with respect to their community profile in soil samples from the three areas. Highly reproducible PCR-DGGE patterns were obtained between triplicate soil samples from all communities evaluated in this study. Although the visual separation of samples from distinct areas was possible, inferences about the clustering were based on the presence and absence matrices obtained based on DGGE profiles, further visualized by NMDS analysis (Fig 2). ANOSIM was used to test for significant segregation of Archaea and Bacteria community patterns (Fig 2, S3 Table). The results for Bacteria and Archaea revealed two distinct groups: one that clustered samples from SV and PC, and another with samples collected in RE (Fig 2a and 2b). In the analyses of ammonia-oxidizing communities, the three areas had separate AOA profiles (Fig 2c), while for AOB bands were found to occur more erratically, not resulting in a clear community segregation, but still allowing the visualization of three partially superimposed groups (Fig 2d).

The correlations between microbial patterns and environmental variables, as determined by RDA, identified the soil properties more strongly related to differences in community profiles (Table 2). Taken together, these results revealed the connection between different community patterns and shifts in the ammonium contents in the evaluated soils—statistically significant for all four evaluated communities (*p* < 0.05). Other soil properties were also related to differences in community patterns, as observed for Mg, B, Cu and silt contents in bacterial communities; B and silt contents in archaeal communities; P and clay contents for AOB communities; and Al, Mn, H+Al and clay contents in AOA communities (Table 2).

### Sequence-based analysis of bacterial and archaeal communities

Two samples from each area were subjected to sequencing, generating a total of 32,221 classified sequences of the 16S rRNA gene– 25,610 sequences were assigned to Bacteria and 6,611 to Archaea—with percentages of 58.0% and 15.2%, respectively.

**Fig 3:**
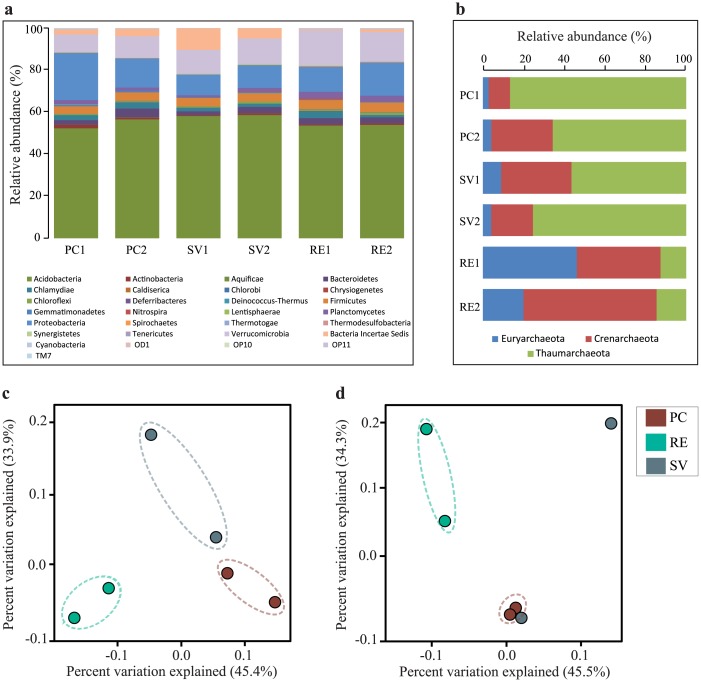
Taxonomic distribution and comparison of bacterial and archaeal communities in soils of three regions of the Atlantic Rainforest by 454-pyrosequencing of the 16S rRNA gene. Percentage of classified sequences affiliated to the phylum level for Bacteria (a) and Archaea (b). Comparison of community composition was conducted by PCoA based on the OTU abundances in each area, and separately for Bacteria (c) and Archaea (d). Values on the axes of panels (c) and (d) indicate the percentage of variance explained on each axis.

The taxonomic composition of bacterial communities encompassed 29 phyla (S4 Table), with most sequences affiliated with the *Acidobacteria* (average of 55.9%), and *Proteobacteria* (17.4%, 10.5% and 14% of the sequences in SV, PC and RE, respectively) (Fig 3a). The taxonomical approach indicated that these phyla occurred in similar proportions across all areas. A major proportion of archaeal sequences was not affiliated to known archaeal phyla (S4 Table). Within the sequences classified into known phyla, we observed the prevalence of those affiliated to *Thaumarchaeota* (51%), *Crenarchaeota* (30%) and *Euryarchaeota* (19%) (Fig 3b). There were differences in the frequency of major taxonomical groups across areas, with a prevalence of *Thaumarchaeota* in PC and SV, while *Crenarchaeota* and *Euryarchaeota* were more frequently found in RE (Fig 3b). The majority of the sequences affiliated to the phylum Thaumarchaeota could not be further classified within known classes—only a few sequences matched to those belonging to the genera *Nitrososphaera* or *Nitrosopumilus*. Sequences affiliated to Euryarchaeota were similar to those from methanogenic archaea, such as the genera *Methanosarcina* and *Methanocella*. All sequences affiliated to the phylum Crenarchaeota were classified as belonging to the class Thermoprotei, not further affiliated into known orders.

**Fig 4:**
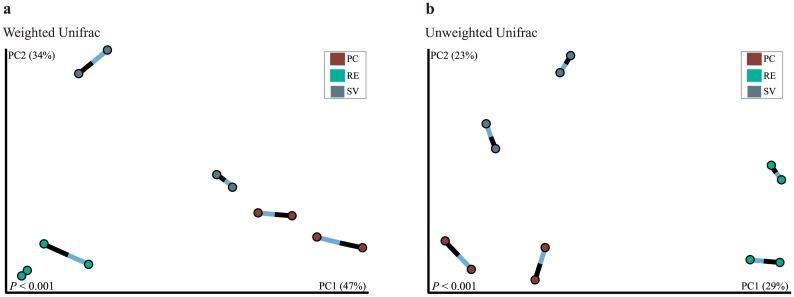
Procrustes analysis based on bacterial and archaeal community assessments on the three areas of the Atlantic Rainforest. Procrustes were run using on Unifrac weighted (a) and unweighted (b) distance matrices. Values on the axes of figures (a) and (b) indicate the percentage of variance explained on each axis.

These differences were assessed in detail on the basis of a taxonomy-independent approach, where sequences were binned into OTUs (determined at 97% of identity levels), and the resulting OTU tables were subjected to a beta-diversity analysis using PCoA. This approach revealed a clear distinction among the communities found in the three assessed areas for bacterial communities (Fig 3c). For the archaeal communities, all sampling areas clustered apart, except for one sample from SV that segregated close to the PC cluster (Fig 3d). Similar patterns for community differences across the areas were observed for Bacteria and Archaea, as indicated by the Procrustes analysis, with M^2^ values of 0.068 and 0.066, using weighted and unweighted UniFrac distances, respectively (Fig 4).

The sequencing data were also used—based on the OTU table—to generate alpha-diversity estimators, which indicated distinctions that were found across the three areas (Table 3). For bacterial analysis, lower values of diversity and richness were found in PC, while similar values were found in SV and RE. For the archaeal analysis, similar diversity and richness values were found across all samples. High coverage of the bacterial and archaeal diversity within the analyzed datasets– 90% and 96% for Bacteria and Archaea, respectively—were found (Table 3).

**Table 3:** Alpha-diversity metrics as determined by observed species, richness, diversity and coverage values in the three distinct areas of the Atlantic Rainforest. Values are mean ± SD; *n* = 2.

	Picinguaba	Santa Virginia	Restinga
*Observed species*			
*Bacteria*	685 ± 32.0	859 ± 1.76	815 ± 16.2
*Archaea*	144 ± 20.9	143 ± 19.2	129 ± 28.8
*Chao1*			
*Bacteria*	1,646 ± 46.8	1,947 ± 34.6	1,950 ± 37.6
*Archaea*	271 ± 2.15	297 ± 13.4	242 ± 9.00
*Shannon*			
*Bacteria*	7.51 ± 0.19	8.23 ± 0.19	7.72 ± 0.12
*Archaea*	5.52 ± 0.57	5.62 ± 0.47	5.36 ± 0.88
*PD*			
*Bacteria*	61.4 ± 0.05	70.0 ± 0.33	72.5 ± 0.54
*Archaea*	8.80 ± 1.00	7.91 ± 0.09	7.50 ± 0.82
*Goods coverage*			
*Bacteria*	0.86 ± 0.005	0.84 ± 0.004	0.83 ± 0.004
*Archaea*	0.89 ± 0.008	0.88 ± 0.011	0.90 ± 0.003

### Analyses of AOA- and AOB-related sequences

**Fig 5:**
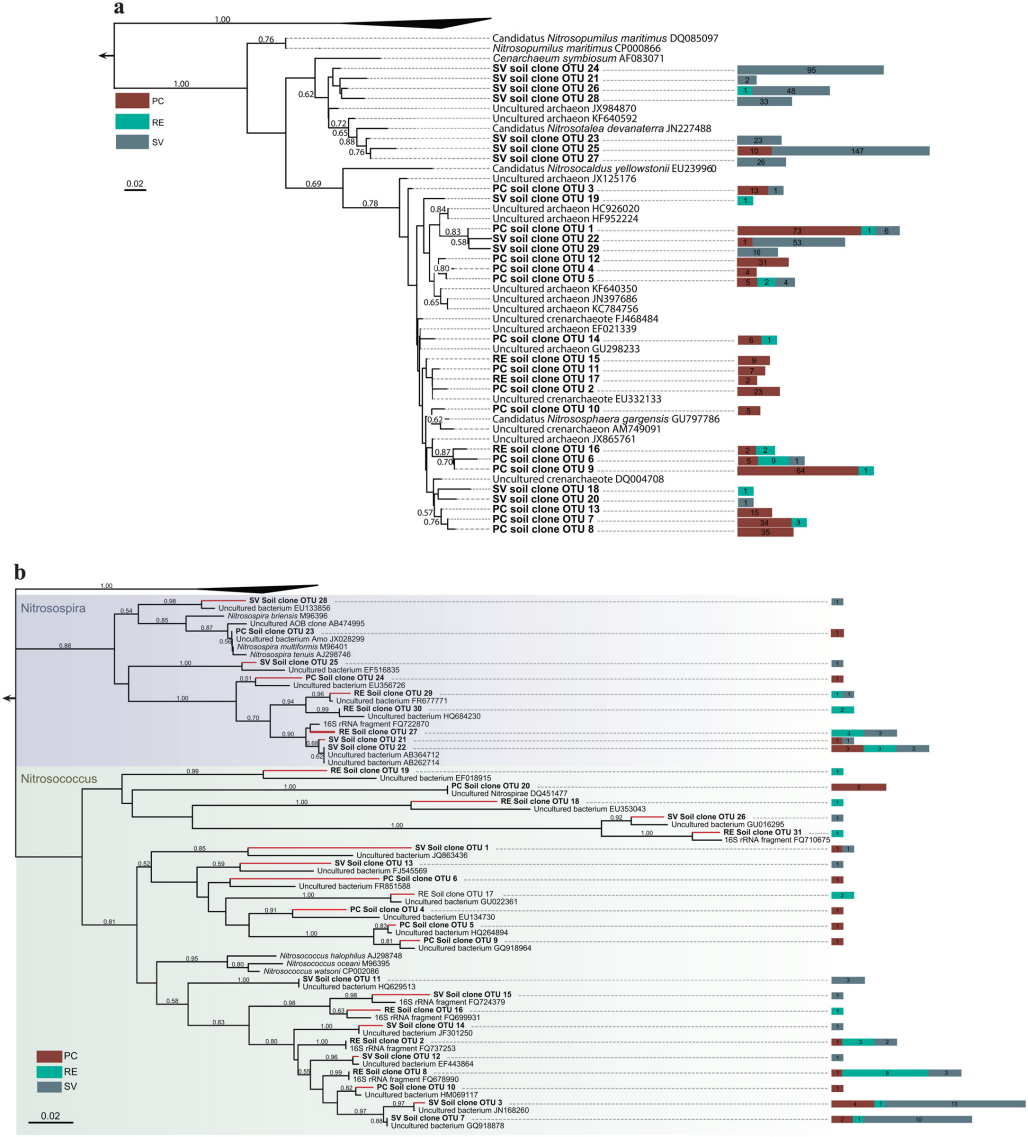
Phylogenetic analysis of 16S rRNA genes with similarities to those from known AOA (a) and AOB (b). The trees display one representative sequence per OTU (unique sequences that shares at least 97% of similarity). Side bars next to OTU labels indicate the number of sequences belonging to the corresponding OTU in each sampled area. Outgroups were made of sequences belonging to AOA for AOB analysis, and vice-versa.

A total of 335, 21 and 456 sequences—binned into 29 OTUs—related to AOA groups were retrieved from soil samples from PC, RE and SV, respectively. These sequences were allocated into a pool of AOA, either from cultured representatives or several uncultivated archaeal sequences, derived from culture-independent analysis of AOA in different environments (Fig 5). It is possible to suggest, based on OTU shared patterns, that distinct groups of AOA were present at the PC and SV sites (Fig 5). Importantly, our taxonomic assignment did not provide the resolution to deeply infer on the likely differential segregation of specific AOA taxa across areas. However, we found a trend for separation of sequences from SV and PC areas, distinctly allocated in branches along our phylogenetic inference (Fig 5). In both groups, most of sequences revealed higher identity with those from unclassified or uncultivated Thaumarchaeota.

Similarly, a total of 98 bacterial sequences were affiliated with known groups of ammonia-oxidizing bacteria– 25, 28 and 45 from PC, RE and SV, respectively. These sequences were binned into 31 OTUs, related to the genera *Nitrosospira* and *Nitrosococcus*, but majorly affiliated with unclassified on uncultivated bacteria (Fig 5). The high diversity observed, together with the lower number of sequences, precluded a proper inference on the distinctions of the members within these communities across the sites.

### Correlations between microbial communities and prevailing characteristics of the sampling sites

The highest correlation values were found—for all fractions of microbial communities—with the vegetation composition in each area. The comparisons based on DGGE data resulted in an R-value of 0.62 for Archaea x plants (either family or genus of plants). For Bacteria, this comparison resulted in values of 0.64 for plant family and 0.63 for plant genus (Table 4). In contrast, R-values for the relationship between microbial communities and chemical or physical attributes of soils were 0.29 or 0.39, respectively, for bacteria and ranged from 0.30 to 0.58 for Archaea (Table 4). For ammonia oxidizers (AOA and AOB), we found a low correlation between chemical and physical attributes and the patterns of both groups, with values between 0.23 and 0.45. However, these communities showed a high correlation with families of plants in the Atlantic Forest, with R-values of 0.97 for AOA and 0.53 for AOB.

**Table 4:** Correlation values, as determined by the Mantel test, between patterns of the microbial communities and characteristics of the Atlantic Rainforest in the sampled areas.

	*Sequence-based*		*DGGE-based*			
Dataset	*Bacteria*	*Archaea*	*Bacteria*	*Archaea*	*AOA*	*AOB*
Chemical data	0.45	0.24	0.29	0.30	0.26	0.23
Physical data	0.78*	0.15	0.39	0.58*	0.45	0.31
Vegetation						
*Family*	*0*.85*	*0*.52*	*0*.64*	*0*.62*	0.97*	0.53*
*Genus*	*0*.61*	*0*.78*	*0*.63*	*0*.62*	ND	ND

ND- not determined

*r ≥0.5 indicates significant correlation

The use of sequences in this approach—as represented by OTUs—resulted in similar results, with values of 0.85 (family) and 0.61 (genus) for comparison with bacterial patterns, and R-values of 0.52 and 0.78 for comparisons between archaeal patterns with plant family and genus, respectively (Table 4).

## Discussion

The Atlantic Rainforest is one of the most diverse biome on Earth [55,56], largely attributed to the high biodiversity of plants and animals. However, the vast majority of the biological diversity in this system comes from the ‘hidden’ microbiomes that permeate this biome. Moreover, this biological resource is known to be responsive to shifts in the environmental conditions, like those observed for complex eukaryotic communities [57]. Thus, a better knowledge on the microbial communities in the Atlantic Rainforest is needed, as well as a better understanding on the mechanisms directly affecting their establishment and dynamics. Given the role of these communities on ecosystem functioning, we argue the importance of the inclusion of the microbial fraction into the inventory of the biodiversity of the Atlantic Rainforest. The knowledge on the magnitude of the microbial diversity in this system is still scarce. For instance, a previous study [58] described that differences in bacterial community diversity and composition can be found between the phyllosphere of individual tree species found in the Atlantic Rainforest. We here added soils to this list, by comparing the bacterial and archaeal communities—apart from AOA and AOB assessments—along an altitudinal gradient. We focused on connecting variations in community composition to differences in soil edaphic characteristics and flora composition.

The soil sites targeted in this study had similar characteristics found across forest soils, such as the typical low fertility, high contents of organic matter, and low pH. These characteristics are also commonly found in other forestry areas, such as in Equatorial Forest in Malaysia [59] and in the Qinling mountains [60]. Importantly, the composition of the vegetation was found to be variable across the sampled areas. As described before [58], shifts in the flora community composition are promoted by differences in microclimate conditions, particularly differences in light incidence and plant phenologies [28]. Here we made use of this argument to build knowledge on how bacterial and archaeal communities of these soil change in accordance with these shifting conditions, and whether different flora compositions influence the assemblages of microbial communities. Given the differences observed in the abundance and composition of the microbiomes, our dataset is suitable to answer some of these questions. Moreover, as the sampled areas are located closely from each other (maximum separation of 95 km), the observed variations are likely to be driven by environmental filtering, as the dispersion limitations of bacteria and archaea would not occur on such spatial scale.

The quantitative values obtained for the community measurements in this study are in line with previous findings. For instance, bacterial abundances in soils are commonly found at the level of 10^9^ per gram of soil [61,62], while this value for archaeal communities is commonly one log scale lower [17]. Variations in this parameter are often related to differences in particular environmental conditions and specific components of the soil microbiome. For example, concerning the ammonia-oxidizing communities, both AOA and AOB presented lower abundances in RE. Although the measured variables could not explain that, the proportional amount of ammonium and nitrate in this area, when compared to SV and PC, indicate the potential limitation of nitrification in RE. It is tempting to speculate that in lower altitudes, the run-off of rains occurs at lower intensity, and thus, the belowground water rise to soil surface more frequently than it occurs at upland areas. Collectively, these may have led to the lower availability of oxygen in this site, thus impairing ammonia oxidation–based metabolism. Indeed, the abundance of AOA was higher than AOB in all three areas, which might be linked to the soil pH, since AOA predominates over AOB under acidic conditions [63–66]. The availability of ammonium is also known to modulate the balance between AOA and AOB [17,19,67], leading to a prevalence of AOA in soils where ammonium is limited [68,69].

Another level of community variations was related to the composition of microbial communities, which in this case tended to be distinct in each analyzed area. Both PCR-DGGE and sequence-based approaches indicated that bacterial, archaeal, and even the ammonia-oxidizing communities were arranged in distinct assemblages along the altitudinal gradient. While PCR-DGGE was efficient to segregate samples between RE and the other two areas, the sequence-based analysis indicated, on the basis of the OTU level, that community compositions were distinct between the three sites. Notably, such spatial variation is commonly found in natural environments, where beta-diversity is often high [3]. The great extension of the Atlantic Rainforest may demand a broader spatial and temporal survey to prove these findings. However, our results, even obtained through a limited number of samples, do provide indications that bacterial and archaeal communities segregate in its composition across the targeted altitudinal gradient.

An additional observation allowed by our sequence-based analysis concerns the microbial groups found in the analyzed areas. The prevalence of *Acidobacteria* and *Proteobacteria* phyla was observed for bacterial communities; and *Thaumarchaeota*, *Crenarchaeota* and *Euryarchaeota* were the named phyla for archaeal communities. Bacterial communities revealed to be composed of taxonomic groups similar to those described in many other soils [70–72], being also similar across all areas. On the other hand, archaeal communities were distinctly assembled in each area at high taxonomical levels (at the phyla classification, for example). Archaeal communities were composed of high proportions of unclassified sequences into any known archaeal phyla. Within those sequences affiliated to specific phyla, a major fraction remained unclassified thereafter, for example, as observed for crenarchaeotal sequences, which were all assigned to unclassified Themoprotei. We tried to improve the affiliation of our sequences by the phylogenetic inference, but mostly of AOA and AOB sequences were better related with unclassified sequences than to those from named groups. Moreover, despite the literature suggests the prevalence of the order Nitrosotaleales as AOA in acidic soils [67], sequences closely affiliated to this order were not abundantly found in our samples. It is tempting to speculate that either these unclassified archaeal sequences may represent new taxa endemic from Atlantic Rainforest soils or they may compose a group of sequences still poorly represented in the available databases. As such, the proper examination of potential taxonomic differences within groups across these areas was technically impaired.

Another question focuses on the discussion of what are the main drivers of such differences found across the spatial scale. We attempted to answer this question using two approaches—RDA-based on DGGE patterns and Mantel tests—linking the characteristics of the sampled areas to the microbial community patterns found in both culture-independent methods. The RDA results was consistent to correlate variations in the contents of ammonium with those in soil microbial communities, although such variations were not statistically validated by univaritate analysis (ANOVA and Tukey test; *p* = 0.162). It shows the importance of using multivariate and correlational analysis to determine major drivers modulating the composition of microbiomes [44]. This result concurs with the findings of the Mantel test, where the composition of the flora had the prevalent correlation with changes in microbial communities. The connection between these results might be based on the systems that plants have to exert selection upon the soil microbes. For instance, the primary rhizosphere effect and the indirect selection made by the litter decomposition—both linked to the release of ammonium in soils [73]. We suggest that the aforementioned role of ammonium variations—derived from plant exudation of litter decomposition—constitutes evidence that the plant selection predominates over other environmental variables on mediating the composition of soil microbiomes in the Atlantic Rainforest. This is in line with a previous study [4] that used neural models to disentangle the contents of organic matter and the contents of base cations (Ca and Mg) as determinant factors influencing microbial diversity in soils. These variables are also tightly linked to plant composition, which is remarkably distinct across these areas. In rainforests, the input of plant materials (by either lettering or rhizosphere) is constant along the year, as opposing to patterns found in temperate or deciduous forests. Moreover, it is worth mentioning that our sampling sites present similar pH values, low amplitude of variation in the average temperature and they are located in a region that has similar rainfall regime. As such, we posit that our inferences despite punctual, may likely be similar to those potentially found in a time series study.

In summary, it can be derived from our findings that the flora composition is the major player in this mosaic, with direct influences on the abundance and composition of the bacterial and archaeal communities in Atlantic Rainforest soils. Collectively, our study contributes to a better understanding of the soil microbiome in this tropical biome, which extends beyond the well-known role of soil pH in tropical forests [74]. We posit that, since variations in pH across all sites did not encompass a large range (i.e. from 3.6 to 3.7), the effect of the plant community composition tended to dominate in the analyzed samples. However, caution is warranted in terms of assuming our findings as absolute and potentially extendable to other systems. We posit that future studies are needed to finely partition the effects of levels of pH and plant diversity and endemicity in Atlantic Rainforest microbiomes. Moreover, as the Atlantic Rainforest harbors high levels of plant endemicity—which exerts selection on distinct phyllosphere microbiomes [58]–the soil microbiomes assembled may also be endemic, either in terms of their components or in their particular assemblages.

## Supporting Information

S1 Table Vegetation composition of the Atlantic Rainforest at the sampling sites [28]. (DOCX)

S2 Table PCR and cycling conditions used to amplify the targeted genes in the present study. (DOCX)

S3 Table Pairwise comparison of similarity (ANOSIM) among microbial communities from in each area (PC, SV and RE), as determined by PCR-DGGE profiles. (DOCX)

S4 Table Percentage of sequences affiliated with bacterial and archaeal phyla in the RDP database. (DOCX)
