# Supplementary material for: Linking the Composition of Bacterial and Archaeal Communities to Characteristics of Soil and Flora Composition in the Atlantic Rainforest
Source: PLoS One. 2016 Jan 11;11(1):e0146566. doi: 10.1371/journal.pone.0146566 (PMC4713446; doi:10.1371/journal.pone.0146566)
Supplement: S1 Table — (DOCX) [file pone.0146566.s001.docx]

**Table S1.** Vegetation composition of the Atlantic Rainforest at the sampling sites [28]

| **Family** | **Genus** | | **Number of arboreal individuals** | | | | | | |  |
| --- | --- | --- | --- | --- | --- | --- | --- | --- | --- | --- |
|  |  |  | Santa Virginia | | Picinguaba | | Restinga | | |  |
| Anacardiaceae | | *Tapirira* sp. | | 0 | | 0 | | 9 | | |
| Annonaceae | | *Guatteria* sp. | | 1 | | 8 | | 100 | | |
| Apocynaceae  Aquifoliaceae | | *Guapira* sp. | | 1 | | 0 | | 0 | | |
|  |  | *Rollinia* sp. | | 0 | | 1 | | 0 |  |  |
|  |  | *Malouetia* sp. | | 0 | | 3 | | 0 |  |  |
|  |  | *Ilex* sp. | | 0 | | 0 | | 24 |  |  |
| Apocynaceae | | *Tabernaemontana* sp. | | 0 | | 4 | | 1 |  |  |
| Araliaceae | | *Dendropanax* sp. | | 0 | | 7 | | 0 |  |  |
|  | | *Oreopanax* sp. | | 0 | | 1 | | 1 |  |  |
| Cardiopteridaceae | | *Citronella* sp. | | 0 | | 6 | | 0 |  |  |
| Caricaceae | | *Jacaratia* sp. | | 0 | | 4 | | 0 |  |  |
| Cecropiaceae | | *Coussapoa* sp. | | 1 | | 0 | | 0 |  |  |
| Celastraceae | | *Maytenus* sp. | | 0 | | 8 | | 48 |  |  |
|  |  | *Salacia* sp. | | 0 | | 7 | | 0 |  |  |
| Chrysobalanaceae | | *Couepia* sp. | | 0 | | 9 | | 2 |  |  |
|  |  | *Hirtella* sp. | | 0 | | 10 | | 2 |  |  |
|  |  | *Licania* sp. | | 5 | | 0 | | 0 |  |  |
| Clusiaceae | | *Calophyllum* sp. | | 0 | | 0 | | 9 |  |  |
|  |  | *Kielmeyera* sp. | | 0 | | 0 | | 29 |  |  |
|  |  | *Garcinia* sp. | | 0 | | 30 | | 28 |  |  |
| Combretaceae | | *Buchenavia* sp. | | 0 | | 1 | | 0 |  |  |
|  |  | *Terminalia* sp. | | 0 | | 3 | | 0 |  |  |
| Cyatheaceae | | *Alsophila* sp. | | 0 | | 2 | | 0 |  |  |
| Sapindaceae | | *Matayba* sp. | | 3 | | 0 | | 0 |  |  |
| Cyatheaceae | | *Cyathea* sp. | | 12 | | 2 | | 1 |  |  |
|  |  | *Alsophila* sp. | | 7 | | 2 | | 0 |  |  |
| Dichapetalaceae | | *Stephanopodium* sp. | | 0 | | 4 | | 0 |  |  |
| Euphorbiaceae | | *Alchornea* sp. | | 30 | | 14 | | 86 |  |  |
|  |  | *Pera* sp. | | 0 | | 0 | | 256 |  |  |
|  |  | *Margaritaria* sp. | | 0 | | 0 | | 5 |  |  |
|  |  | *Hyeronima* sp. | | 1 | | 0 | | 4 |  |  |
|  |  | *Euphorbiaceae* sp. | | 0 | | 1 | | 0 |  |  |
|  |  | *Mabea* sp. | | 0 | | 1 | | 0 |  |  |
| Elaeocarpaceae | | *Slonea* sp. | | 0 | | 0 | | 8 |  |  |
| Erythroxylaceae | | *Erythroxylum* sp. | | 0 | | 1 | | 0 |  |  |

| Euphorbiaceae | *Croton* sp. | 2 | 0 | 0 |
| --- | --- | --- | --- | --- |

|  | *Pausandra* sp. | 0 | 16 | 0 |
| --- | --- | --- | --- | --- |
|  | *Sapium* sp. | 0 | 2 | 0 |
| Elaeocarpaceae | *Sloanea* sp. | 0 | 18 | 0 |
| Fabaceae | *Andira* sp. | 0 | 0 | 37 |
|  | *Copaifera* sp. | 0 | 3 | 0 |
|  | *Dalbergia* sp. | 0 | 0 | 1 |
|  | *Dahlstedtia* sp. | 0 | 2 | 0 |
|  | *Hymenaea* sp. | 0 | 1 | 0 |
|  | *Inga* sp. | 2 | 6 | 29 |
|  | *Myrocarpus* sp | 0 | 1 | 0 |
|  | *Ormosia* sp. | 0 | 0 | 2 |
|  | *Plastymiscium* sp. | 0 | 1 | 0 |
|  | *Swartzia* sp. | 0 | 15 | 4 |
|  | *Tachigali* sp. | 0 | 10 | 0 |
|  | Zollernia sp. | 0 | 2 | 0 |
| Icacinaceae | *Citronella* sp. | 3 | 0 | 0 |
| Lacistemaceae | *Lacistema* sp. | 0 | 0 | 15 |
| Lamiaceae | *Aegiphila* sp. | 0 | 2 | 0 |
| Lauraceae | *Endlicheria* sp. | 0 | 0 | 8 |
|  | *Cryptocarya* sp. | 2 | 9 | 0 |
|  | *Nectandra* sp. | 0 | 0 | 29 |
|  | *Licaria* sp. | 0 | 6 | 0 |
|  | *Ocotea* sp. | 4 | 10 | 1 |
|  | *Rhodostemonodaphne* sp. | 0 | 1 | 0 |
| Lecythidaceae | *Cariniana* sp. | 0 | 4 | 0 |
| Malpighiaceae | *Byrsonima* sp. | 0 | 0 | 6 |
| Melastomataceae | *Miconia* sp. | 3 | 1 | 34 |
|  | *Miconia* sp. | 0 | 1 | 0 |
|  | *Mollinedia* sp. | 1 | 94 | 0 |
|  | *Tibouchina* sp. | 1 | 0 | 1 |
| Magnoliaceae | *Magnólia* sp. | 0 | 1 | 0 |
| Malvaceae | *Eriotheca* sp. | 0 | 7 | 0 |
|  | *Quararibea* sp. | 0 | 7 | 0 |
| Meliaceae | *Trichilia* sp. | 2 | 6 | 0 |
|  | *Cabralea* sp. | 1 | 2 | 0 |

|  | *Guarea* sp. | 4 | 2 | 67 |
| --- | --- | --- | --- | --- |
| Monimiaceae | *Miconia* sp. | 1 | 1 | 0 |
|  | *Mollinedia* sp. | 8 | 94 | 0 |
| Moraceae | *Brosimum* sp. | 0 | 11 | 0 |
|  | *Ficus* sp. | 0 | 5 | 1 |
|  | *Sorocea* sp. | 0 | 20 | 0 |
| Myristicaceae | *Virola* sp. | 0 | 15 | 0 |

| Myrsinaceae | *Myrsine* sp. | 0 | 0 | 48 |
| --- | --- | --- | --- | --- |
|  | *Gomidesia* sp. | 0 | 0 | 97 |
|  | *Ardisia* sp. | 0 | 5 | 0 |
|  | *Rapanea* sp. | 2 | 1 | 0 |
| Myrtaceae | *Eugenia* sp. | 2 | 105 | 78 |
|  | *Campomanesia* sp | 1 | 8 | 1 |
|  | *Marlierea* sp. | 2 | 40 | 35 |
|  | *Psidium* sp. | 0 | 0 | 16 |
|  | *Calyptranthes* sp. | 3 | 18 | 15 |
|  | *Myrcia* sp. | 7 | 40 | 227 |
|  | *Myrciaria* sp. | 0 | 10 | 0 |
|  | *Plinia* sp. | 0 | 1 | 0 |
| Nyctaginaceae | *Guapira* sp. | 12 | 39 | 29 |
| Polygonaceae | *Coccoloba* sp. | 0 | 0 | 8 |
| Phyllanthaceae | *Hieronyma* sp. | 1 | 6 | 0 |
| Piperaceae | *Piper* sp. | 1 | 0 | 0 |
| Proteaceae | *Euplassa* sp. | 0 | 0 | 32 |
|  | *Roupala* sp. | 0 | 2 | 3 |
| Rosaceae | *Prunus* sp. | 0 | 0 | 8 |
| Rubiaceae | *Amaioua* sp. | 0 | 0 | 8 |
|  | *Alseis* sp. | 0 | 8 | 0 |
|  | *Bathysa*sp. | 7 | 70 | 0 |
|  | *Chomelia* sp. | 1 | 0 | 0 |
|  | *Coussarea* sp. | 0 | 102 | 0 |
|  | *Faramea* sp. | 0 | 7 | 0 |
|  | *Faramea* sp. | 0 | 0 | 12 |
|  | *Ixora* sp. | 0 | 1 | 0 |
|  | *Genipa* sp. | 0 | 0 | 4 |

|  | *Posoqueria* sp. | 0 | 1 | 0 |
| --- | --- | --- | --- | --- |
|  | *Rubiaceae* sp. | 0 | 1 | 0 |
|  | *Rudgea* sp. | 0 | 6 | 0 |
|  | *Rustia* sp. | 1 | 44 | 0 |
| Rutaceae | *Zanthoxylum* sp. | 0 | 1 | 6 |
| Olacaceae | *Heisteria* sp. | 1 | 1 | 0 |
| Ochnaceae | *Ouratea* sp. | 0 | 2 | 0 |
| Olacaceae | *Tetrastylidium* sp. | 0 | 14 | 0 |
| Salicaceae | *Casearia* sp. | 1 | 0 | 0 |
| Sapindaceae | *Matayba* sp. | 3 | 0 | 0 |
|  | *Cupania* sp. | 1 | 1 | 0 |
| Sapotaceae | *Chrysophyllum* sp. | 0 | 45 | 0 |
|  | *Ecclinusa* sp. | 0 | 10 | 0 |
|  | *Pouteria* sp. | 2 | 0 | 0 |
|  | *Chrysophyllum* sp. | 1 | 0 | 0 |
| Solanaceae | *Solanaceae* sp.. | 0 | 1 | 0 |
|  | *Solanum* sp. | 2 | 0 | 0 |
| Theophrastaceae | *Clavija* sp. | 0 | 0 | 2 |
| Thymelaeaceae | *Daphnopsis* sp. | 2 | 0 | 0 |
| Urticaceae | *Cecropia* sp. | 1 | 9 | 2 |
|  | *Coussapoa* sp. | 0 | 4 | 0 |
|  | *Vitex* sp. | 0 | 0 | 4 |
