# Supplementary material for: Linking the Composition of Bacterial and Archaeal Communities to Characteristics of Soil and Flora Composition in the Atlantic Rainforest
Source: PLoS One. 2016 Jan 11;11(1):e0146566. doi: 10.1371/journal.pone.0146566 (PMC4713446; doi:10.1371/journal.pone.0146566)
Supplement: S2 Table — (DOCX) [file pone.0146566.s002.docx]

**Table S2.** PCR mixtures, primers and cycling conditions used to amplify the target genes

| ***Primers quantitative PCR*** | ***Primer sequence (5’-3’)*** | ***Thermal cycling conditions*** |
| --- | --- | --- |
| **Bacteria (16S rRNA)** | | |
| P1  P2 (Muyzer et al., 1993) | ACGGGGGGCCTACGGGAGGCAGCAG  ATTACCGCGGCTGCTGG | 95°C 3 min, 1 cycle  94ºC 30 s, 55ºC 30 s, 72ºC 30 s, 35 cycles |
| **Archaea (16S rRNA** | |  |
| 340F  1000R (Gantner et al. 2011) | CCCTAYGGGGYGCASCAG  GGCCATGCACYWCYTCTC | 98°C 2 min, 1 cycle  94ºC 30 s, 57ºC 30 s, 72ºC 1 min 30 s, 35 cycles |
| **amoA (AOA)** |  | |
| Amo23F (Tourna et al., 2008)  CrenamoA616r48x (Nicol et al., 2008) | ATGGTCTGGCTWAGACG  GCCATCCABCKRTANGTCCA | 95°C 3 min, 1 cycle  95ºC 15 s, 55ºC 40 s, 72ºC 30 s, 35 cycles |
| **amoA (AOB)** | | |
| CTO189fA/CTO189fB  CTO189fC  CTO654r (Kowalchuk et al., 1997)  R1 (Hermansson and Lindgren, 2001) | GGAGRAAAGCAGGGGATCG GGAGRAAAGCAGGGGATCG  GGAGGAAAGTAGGGGATCG CTAGCYTTGTAGTTTCAAACGC  CGTCCTCTCAGACCARCTACTG | 95°C 3 min, 1 cycle  95ºC 30 s, 58ºC 1 min, 68ºC 45 s, 35 cycles |
| ***Primers DGGE*** | ***Primer sequence (5’-3’)*** | ***Thermal cycling conditions*** |
| **Bacteria (16S rRNA)** | | |
| U968┴CG  R1378 (Heuer et al., 1997) | CGCCCGGGGCGCGCCCCGGGCGGGGCGGGGGCACG  CGGTGTGTACAAGGCCCGGGAACG | 94°C 4 min, 1 cycle  94ºC 1 min, 56ºC 1 min, 72ºC 2 min, 35 cycles  72ºC 10 min of final extension |
| **Archaea (16S rRNA)** | | |
| Arch21F  Arch958R (Øvreas et al., 1997) | TTCCGGTTGATCCYGCCGGA  YCCGGCGTTGAMTCCAATT | 95°C 5 min, 1 cycle  95ºC 30 s, 53ºC 30 s, 72ºC 1 min, 30 cycles  72ºC 6 min of final extension |
| Arch340F┴GC  Arch519R (Øvreas et al., 1997 | CTACGGGGYGCASCAG  TTACCGCGGCKGCTG | 95°C 5 min, 1 cycle  95ºC 30 s, 53ºC 30 s, 72ºC 1 min, 30 cycles  72ºC 6 min of final extension |
| **amoA (AOA)** | | |
| crenamA23f  crenamoA616r (Tourna et al., 2008) | ATGGTCTGGCTWAGACG  GCCATCCATCTGTATGTCCA | 95°C 3 min, 1 cycle  95ºC 15 s, 55ºC 40 s, 72ºC 30 s, 35 cycles  72ºC 10 min of final extension |
| **amoA (AOB)** | |  |
| CTO189f  CTO654r (Kowalchuk et al.,1997) | GGCACGGGGGGAGRAAAGYAGGGGATCG  CTAGCYTTGTAGTTTCAAACGC | 95°C 3 min, 1 cycle  95ºC 30 s, 58ºC 1 min, 68ºC 45 s, 35 cycles  68ºC 5 min of final extension |
| ***Primers pyrosequencing*** | ***Primer sequence (5’-3’)*** | ***Thermal cycling conditions*** |
| **Bacteria (16S rRNA)** | | |
| 520F | GGGAYTYDTGNGAAA | 95°C 3 min, 1 cycle  95ºC 45 s, 57ºC 1 min 45 s, 72ºC 1 min, 30 cycles  72ºC 4 min of final extension |
| 802R (RDP Database) | GGTTACCRGHTCTAATCC  TAATACGACAGTTTCATC  CTACTAATCCDSRGGTMT  TAATACNVGATCGGTTCC |  |
| **Archaea (16S rRNA)** | | |
| ArcF | AYTGGGYDTAAAGNG | 95°C 5 min, 1 cycle  95ºC 30 s, 55.8ºC 30 s, 72ºC 1 min, 30 cycles  72ºC 10 min of final extension |
| ArcR (Jesus et al., 2010) | TACCRGGGTHTCTAATCC  TACCAGAGTATCTAATTC  CTACDSRGGTMTCTAATC  TACNVGGGTATCTAATCC |  |
