# Supplementary material for: Linking the Composition of Bacterial and Archaeal Communities to Characteristics of Soil and Flora Composition in the Atlantic Rainforest
Source: PLoS One. 2016 Jan 11;11(1):e0146566. doi: 10.1371/journal.pone.0146566 (PMC4713446; doi:10.1371/journal.pone.0146566)
Supplement: S3 Table — (DOCX) [file pone.0146566.s003.docx]

**Table S3.** One-way ANOSIM showing similarity between sampling sites based on DGGE community profiles

| Sites | *Bacteria* | *Archaea* | *AOB* | *AOA* |
| --- | --- | --- | --- | --- |
| **Santa Virginia**  **x**  **Picinguaba** | 0.35 | 0.73* | 0.66* | 1.0** |
| **Santa Virginia**  **x**  **Restinga** | 0.80** | 0.87** | 0.68* | 1.0** |
| **Picinguaba**  **x**  **Restinga** | 0.91** | 0.75* | 0.77** | 1.0** |

R values are expressed. R> 0.75 are statistically different; > 0.5 have overlapping but are still clearly different; and <0.5 do not show statistical difference.

*Statistically different value (> 0.5)

**Statistically different value (> 0.75)
