# Supplementary material for: Linking the Composition of Bacterial and Archaeal Communities to Characteristics of Soil and Flora Composition in the Atlantic Rainforest
Source: PLoS One. 2016 Jan 11;11(1):e0146566. doi: 10.1371/journal.pone.0146566 (PMC4713446; doi:10.1371/journal.pone.0146566)
Supplement: S4 Table — (DOCX) [file pone.0146566.s004.docx]

**Table S4.** Percentage of sequences affiliated with each phylum for both domains based on analysis in RDP database.

|  | **Sampling site** | | | | | |
| --- | --- | --- | --- | --- | --- | --- |
|  | **Picinguaba** | | **Santa Virginia** | | **Restinga** | |
| ***Phylum*** | *Sample 1* | *Sample 2* | *Sample 1* | *Sample 2* | *Sample 1* | *Sample 2* |
| **Archaeal 16S rRNA** | **%** | | | | | |
| Euryarchaeota | 0.3 | 0.2 | 0.3 | 0.2 | 2.0 | 0.8 |
| Crenarchaeota | 1.2 | 1.6 | 1.0 | 0.8 | 1.8 | 2.5 |
| Thaumarchaeota | 9.7 | 3.5 | 1.7 | 3.0 | 0.5 | 0.5 |
| Unclassified | 88.8 | 94.7 | 97.0 | 96.0 | 95.7 | 96.2 |
| **Bacterial 16S rRNA** |  | | | | | |
| Actinobacteria | 0.3 | 0.7 | 1.4 | 0.6 | 0.4 | 0.5 |
| Bacteroidetes | 1.6 | 2.7 | 1.6 | 3.8 | 2.6 | 2.7 |
| Chlamydiae | 1.3 | 1.2 | 1.5 | 2.1 | 2.1 | 0.7 |
| Planctomycetes | 0.3 | 0.7 | 0.8 | 0.6 | 1.5 | 1.1 |
| Verrucomicrobia | 11.1 | 11.6 | 7.8 | 9.9 | 16.1 | 13.5 |
| Firmicutes | 0.4 | 0.3 | 0.3 | 0.6 | 0.7 | 0.6 |
| Chloroflexi | 9 | 3.3 | 1.3 | 2.4 | 0.1 | 0.2 |
| Acidobacteria | 57.1 | 57 | 51.1 | 55.3 | 53.1 | 53.3 |
| Proteobacteria | 7.1 | 7.9 | 16 | 10.4 | 7.2 | 11.1 |
| Unclassified | 11.8 | 17.9 | 18.2 | 14.3 | 16.2 | 16.3 |
